# Transcription Factor NRF2 Participates in Cell Cycle Progression at the Level of G1/S and Mitotic Checkpoints

**DOI:** 10.3390/antiox11050946

**Published:** 2022-05-11

**Authors:** Diego Lastra, Maribel Escoll, Antonio Cuadrado

**Affiliations:** 1 Department of Biochemistry, Medical College, Autonomous University of Madrid (UAM), 28029 Madrid, Spain; diegolastra@iib.uam.es (D.L.); mescoll@iib.uam.es (M.E.); 2 Instituto de Investigaciones Biomédicas “Alberto Sols” (CSIC-UAM), C/Arturo Duperier, 4, 28029 Madrid, Spain; 3 Instituto de Investigación Sanitaria La Paz (IdiPaz), C/de Pedro Rico, 6, 28029 Madrid, Spain; 4 Centro de Investigación Biomédica en Red de Enfermedades Neurodegenerativas (CIBERNED), C/Valderrebollo, 28031 Madrid, Spain

**Keywords:** cell division cycle, restriction point, check point, NRF2

## Abstract

Transcription factor NRF2 is a master regulator of the multiple cytoprotective responses that confer growth advantages on a cell. However, its participation in the mechanisms that govern the cell division cycle has not been explored in detail. In this study, we used several standard methods of synchronization of proliferating cells together with flow cytometry and monitored the participation of NRF2 along the cell cycle by the knockdown of its gene expression. We found that the NRF2 levels were highest at S phase entry, and lowest at mitosis. NRF2 depletion promoted both G1 and M arrest. Targeted transcriptomics analysis of cell cycle regulators showed that NRF2 depletion leads to changes in key cell cycle regulators, such as *CDK2*, *TFDP1*, *CDK6*, *CDKN1A* (p21), *CDKN1B* (p27), *CCNG1*, and *RAD51*. This study gives a new dimension to NRF2 effects, showing their implication in cell cycle progression.

## 1. Introduction

NRF2 (Nuclear factor (erythroid–derived 2)–like 2), encoded by the gene *NFE2L2*, is a transcription factor that belongs to the basic region leucine zipper (bZIP) family. It regulates the expression of over 250 genes through its heterodimerization with MAF proteins and interaction with its target sequence, the Antioxidant Response Element (ARE) [1]. NRF2 activates the transcription of cytoprotective genes in response to oxidative damage, electrophiles, xenobiotics, or pro–inflammatory stimuli, and is considered a master regulator of homeostatic processes, including cell detoxification, antioxidant cell defense, metabolism regulation, control of proteostasis, inflammation resolution, DNA repair, cell survival, etc. [2,3].

Due to the plethora of cytoprotective and homeostatic responses elicited by NRF2, much interest has been shown in its implications for cancer initiation and progression [4]. Not surprisingly, many studies have reported pro–tumoral effects associated with these cytoprotective functions, including ROS scavenging [5], chemoresistance and radioresistance [6,7,8], metabolic reprogramming towards anabolic pathways and the pentose phosphate pathway [9], or evasion of apoptosis [10,11]. Other studies have directly linked NRF2 to key pro–tumoral pathways such as KRAS, mutant p53, the Hippo pathway, or WIP [12,13,14,15].

However, the impact of NRF2 on cell cycle progression and cell redistribution in the cell cycle phases has not been explored [16]. Cell transit throughout the G1, S, G2, and M cycle phases is governed by a protein regulatory network of cyclin–dependent kinases (CDKs) [17], whose activity is controlled by association with various phase–specific cyclins, regulatory phosphorylation, and binding of CDK inhibitors [18]. Together, these molecular engines propel cell proliferation through several checkpoints that ensure proper progression along the cell cycle according to mass cell growth and DNA replication, integrity, and division. In this study, we analyzed the effect of NRF2 throughout the cell cycle phases under standard proliferating conditions in cell cultures and identified correlations with the changes in key cell cycle regulators, such as *CDK2*, *TFDP1*, *CDK6*, *CDKN1A* (p21), *CDKN1B* (p27), *CCNG1*, and *RAD51*. This study gives a new layer to the characterization of the role of NRF2 in cell cycle progression.

## 2. Materials and Methods

### 2.1. Cell Culture, Reagents and Synchronization

Validated U–373 MG, U–87 MG, and MDA–MB–231 cell lines were grown in Dulbecco’s Modified Eagle Medium (DMEM), supplemented with 10% fetal bovine serum (FBS), 4 mM L–glutamine (Gibco ID 25030081, Waltham, MA, USA), 80 mg/mL gentamicin (Normon Laboratories, Tres Cantos, Spain), and 1% amphotericin B solution (Lonza ID 17–836E, Basel, Switzerland), in 5% CO_2_ at 37 °C. Sulforaphane (SFN) was purchased from LKT Laboratories (ID S8044, St Paul, MN, USA). Paraquat (PQ) was purchased from Sigma Aldrich (ID M2254, St. Louis, MO, USA). For synchronization at G1/G0, cells were FBS–deprived for 64 h and then replated in a standard medium (0 h point). For synchronization before S entry, a double thymidine pulse was performed. Cells were treated with 2.5 mM thymidine (Abcam, ID ab143719, Cambridge, UK), and then washed with PBS twice and maintained in thymidine–free medium for 8 h. Then, a second thymidine pulse was performed under the same conditions, and cells were finally chased to standard medium (0 h point). For synchronization in the M phase, cells were treated with 75 ng/mL nocodazole (Sigma Aldrich, ID M1404) for 16 h. Then, round and partially detached cells were harvested and plated in a nocodazole–free medium (0 h point).

### 2.2. Lentiviral Vector Production, and Infection

HEK293T cells were transiently co–transfected with 6 μg of pSPAX2 packaging plasmid (12260, Addgene, Watertown, MA, USA) and 6 μg of VSV–G envelope protein plasmid pMD2G (12259, Addgene) and 10 μg of the corresponding lentiviral vector, using polyethyleneimine (PEI) (Polyscience, ID 23966–1, Niles, IL, USA), to produce the pseudotyped lentiviral particles. The following lentiviral vectors were used: lentiviral vector shRNA control (shco) (1864, Addgene); and shNRF2–1 (NM_006164 TRCN0000273494) and shNRF2–2 (NM_006164 TRCN0000007555) which were both were purchased from Sigma-Aldrich (MISSION shRNA). Lentiviral vector pWPXL–NRF2–ΔETGE (NRF2^ΔETGE^) and pWPXL–NRF2–dN (NRF2–dN) were homemade using pWPXL as expression vector (control) (12257, Addgene). Cells were infected in the presence of 4 μg/mL polybrene (Sigma-Aldrich, ID TR–1003) and selected with 1 μg/mL puromycin (Sigma-Aldrich, ID P8833).

### 2.3. Immunoblot

This protocol was essentially performed as in [13]. Briefly, cells were homogenized in lysis buffer (50 mM Tris pH 7.6, 400 mM NaCl, 1 mM EDTA, 1 mM EGTA, and 1% SDS), denaturalized at 95 °C for 15 min, sonicated, and pre–cleared by centrifugation. Twenty µg of protein for each sample was resolved through SDS–PAGE and then transferred to Immobilon–P (Millipore) membranes. The following antibodies were used: NRF2 (homemade, validated in [19]); HO–1 (homemade, validated in [20]); Cyclin B (BD Biosciences, ID 554177, Franklin Lakes, NJ, USA); GAPDH (Merck Millipore, ID CB1001, Burlington, MA, USA); p21 (Santa Cruz Biotechnology, ID sc–6246, Dallas, TX, USA); p27 (BD Biosciences, ID 610241); RAD51 (Thermo Fisher Scientific, ID PA5–27195, Waltham, MA, USA); CDK2 (Santa Cruz Biotechnology, ID sc–6248). Adequate peroxidase–conjugated secondary antibodies were used for protein detection by enhanced chemiluminescence (Thermo Fisher Scientific, ID 12644055).

### 2.4. Analysis of mRNA Levels

Total RNA extraction and qRT–PCR were completed, as detailed in [13]. Primer sequences are shown in Table 1. Data were analyzed using the ΔΔCT method. Raw data were normalized using the geometric mean of the housekeeping genes *GAPDH* and *TBP*. All PCRs were performed from quadruplicate samples.

### 2.5. MTT and Cell Growth Assays

Cells were treated with thiazolyl blue tetrazolium bromide (MTT) (Sigma Aldrich, ID M5655) for 1 h, then the medium was retrieved, and the cells were directly lysed in DMSO for 30 min. One hundred µL of the supernatants were analyzed in 96–well multiwell plates at 550 nm in a VERSAmax microplate reader (Molecular Devices, San Jose, CA, USA). For cell growth kinetics, cells were counted in a Neubauer improved Bright–Line counting chamber (Marienfeld) using Trypan Blue solution (Sigma Aldrich, ID T8154) to discriminate the dead cells.

### 2.6. Flow Cytometry

For analysis of DNA content, the cells were harvested, pelleted, and fixed in cold 70% ethanol diluted in PBS for at least 24 h at −20 °C. Ethanol was then removed by centrifugation (1100 rpm, 5 min) and cells were washed twice with PBS containing 0.03% Triton–X100 (PBST) (Sigma Aldrich, ID 9002-93-1). Finally, cells were resuspended in PBST with 1µg/mL Hoechst 33342 (Thermo Fisher Scientific, ID H3570) and incubated for 30 min in darkness. Analysis of the DNA content was performed with a FACSCanto II (BD Biosciences) flow cytometer in at least 10,000 events per condition. Calculation of the number of cells in G1, S, and G2 was completed using the Modfit (version 5.0) software. For EdU analysis, the Click–iT™ EdU Alexa Fluor™ 647 Flow Cytometry Assay Kit (Molecular Probe, ID C10424) was used, according to the manufacturer’s instructions. Briefly, cells were treated with 10 µM EdU for 1 h, and then harvested, fixed, and permeabilized. For analysis of DNA content, cells were counterstained with 1µg/mL Hoechst 33342 (Thermo Fisher Scientific, ID H3570) for 30 min in darkness. Analysis of EdU incorporation and DNA content was performed with a FACSCanto II (BD Biosciences) flow cytometer, analyzing at least 10,000 events per condition. For analysis of intracellular reactive oxygen species (ROS), cells were incubated for 1 h at 37 °C with 2 μM hydroethidine (HE) (Thermo Fisher Scientific, ID D11347) in the dark, then detached from the plate, washed once with cold PBS, and analyzed immediately. Oxidized HE fluorescence (Excitation/Emission: 518/606 nm) was analyzed in a CytoFLEX S Flow Cytometer (Beckman Coulter, Brea, CA, USA).

### 2.7. RT^2^ Profiler PCR Array of Human Cell Cycle

RNA samples were extracted using TRIzol (Invitrogen, ID 15596026, Waltham, MA, USA), according to the manufacturer’s instructions. Five hundred ng of each mRNA sample were retrotranscribed using the RT^2^ First Strand kit (QUIAGEN, ID 330401, Hilden, Germany). Transcript levels for 84 different cell cycle–related genes were analyzed on an RT^2^ Profiler™ PCR Array Human Cell Cycle, from QUIAGEN (ID 330231, GeneGlobe ID PAHS–020Z; https://geneglobe.qiagen.com/us/product-groups/rt2-profiler-pcr-arrays, accessed on 8 May 2022), according to the manufacturer’s instructions, using RT2 SYBR Green/ROX qPCR Master Mix (QUIAGEN, ID 330522) for the PCR reaction. The PCR reaction was performed on a 7900 HT Fast Real–Time PCR System (Applied Biosystems, Waltham, MA, USA). Analysis of gene expression changes was performed, according to the manufacturer’s recommendations, using the ΔΔCT method. Genes analyzed were the following: *ABL1*; *ANAPC2*; *ATM*; *ATR*; *AURKA*; *AURKB*; *BCCIP*; *BCL2*; *BIRC5*; *BRCA1*; *BRCA2*; *CASP3*; *CCNA2*; *CCNB1*; *CCNB2*; *CCNC*; *CCND1*; *CCND2*; *CCND3*; *CCNE1*; *CCNF*; *CCNG1*; *CCNG2*; *CCNH*; *CCNT1*; *CDC16*; *CDC20*; *CDC25A*; *CDC25C*; *CDC34*; *CDC6*; *CDK1*; *CDK2*; *CDK4*; *CDK5R1*; *CDK5RAP1*; *CDK6*; *CDK7*; *CDK8*; *CDKN1A*; *CDKN1B*; *CDKN2A*; *CDKN2B*; *CDKN3*; *CHEK1*; *CHEK2*; *CKS1B*; *CKS2*; *CUL1*; *CUL2*; *CUL3*; *E2F1*; *E2F4*; *GADD45A*; *GTSE1*; *HUS1*; *KNTC1*; *KPNA2*; *MAD2L1*; *MAD2L2*; *MCM2*; *MCM3*; *MCM4*; *MCM5*; *MDM2*; *MKI67*; *MNAT1*; *MRE11A*; *NBN*; *RAD1*; *RAD17*; *RAD51*; *RAD9A*; *RB1*; *RBBP8*; *RBL1*; *RBL2*; *SERTAD1*; *SKP2*; *STMN1*; *TFDP1*; *TFDP2*; *TP53*; *WEE1*.

## 3. Results

### 3.1. NRF2 Alters Cell Cycle Progression and Proliferation

**Figure 1:**
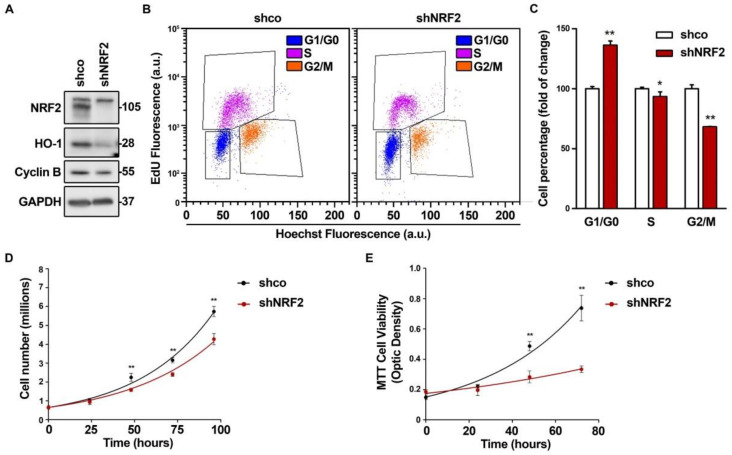
NRF2 deficiency alters cell cycle distribution and proliferation rate. U–373 MG cells were transduced with lentiviral vectors containing control (shco) or shNRF2–1 (shNRF2). (**A**) Representative immunoblot analysis of NRF2, HO–1, Cyclin B, and GAPDH as a loading control; (**B**) Changes in cell cycle distribution between G1, S, and G2/M phases were determined by combined EdU and Hoechst staining (a.u., arbitrary units of fluorescence emission). A representative sample of 10,000 cells is shown for each condition; (**C**) Flow cytometry analysis of changes in cell cycle distribution for G1, S, and G2/M phases in EdU/Hoechst–stained cells. Data are presented as mean ± S.D. ** *p* ≤ 0.01; * *p* ≤ 0.05 vs. shco according to a Student’s *t*-test (*n* = 3); (**D**) Proliferation rates of shco and shNRF2 cells were determined by trypan blue staining. The curve was fitted according to an exponential growth model with the least square fit. Data are presented as mean ± S.D. ** *p* ≤ 0.01 vs. shco according to a two–way ANOVA test (*n* = 3); (**E**) Proliferation rate in shNRF2 cells based on the MTT metabolic assay. The curve was fitted according to an exponential growth model with the least square fit. Data are presented as mean ± S.D. ** *p* ≤ 0.01 vs. shco according to a two–way ANOVA test (*n* = 4).

To determine the relevance of NRF2 in cell cycle progression, we knocked down NRF2 in U–373 MG glioblastoma–derived cells (Figure 1) and in two unrelated cell lines, U–87 MG (Figure S1A–C) and MDA–MB–231 (Figure S1D–F), that yielded similar results. As shown in Figure 1A, after 3 days of interference, we confirmed a drastic reduction of NRF2 protein levels, and of its target HO–1, used as a control of NRF2 transcriptional activity. There was also a reduction in the G2/M phase marker Cyclin B. Flow cytometry analysis of cell cycle phase distribution was performed in the cells labeled with EdU, which identifies S–phase cells, together with Hoechst 33342, which gives a quantitative measurement of the DNA content (Figure 1B,C). The quantification of EdU–negative cells indicated that the NRF2 knockdown increased G1/G0 and decreased the S and G2/M cell numbers. The analysis of the Hoechst 33342–stained cells without EdU staining, with the help of the Modfit software, yielded similar results (Figure S2A,B) and was used in subsequent experiments to analyze the cell cycle phase distribution. Similar results were obtained with another shRNA for NRF2 knock–down (Figure S2A–D), and with a dominant–negative NRF2 mutant lacking the transactivation domain (Figure S2E,F) [21]. Together, these results indicate that the redistribution of the cells towards G1/G0 is a consequence of the NRF2 depletion. The cell redistribution in the G0/G1 and G2/M phases correlated with a change in the proliferation rate, as determined by cell counts (Figure 1D) and metabolic activity (Figure 1E) along 4 days of growth. This effect was also observed in the U–87 MG and MDA–MB–231 cells (Figure S1C,F). In conclusion, the absence of NRF2 impairs cell cycle phase distribution and proliferation rate.

**Figure 2:**
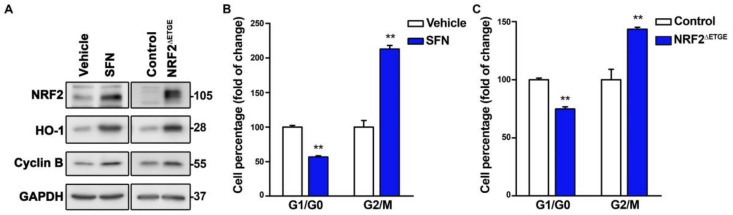
NRF2 activation propels the proliferation rate. U–373 MG glioblastomas cells were treated with vehicle or 10 µM SFN for 16 h, or transduced with a lentiviral vector expressing mutant NRF2^ΔETGE^ or empty vector (Control). (**A**) Representative immunoblot analysis of NRF2, HO–1, Cyclin B, and GAPDH as a loading control; (**B**,**C**) Flow cytometry analysis of G1/G0, and G2/M cell distribution in Hoechst–stained cells. Data are presented as mean ± S.D. ** *p* ≤ 0.01 according to a Student’s *t*-test (*n* = 3).

We next tested if chemical or genetic NRF2 activation might exert the opposite effect. We treated U–373 MG cells with the NRF2 activator sulforaphane (SFN) and, considering that NRF2 activators might have off–target effects, we also used a lentiviral vector encoding the highly active NRF2^ΔETGE^ mutant, which is partially resistant to proteasomal degradation due to loss of interaction with its main repressor KEAP1 [1]. In both cases, we observed the expected increase in both the NRF2 and HO–1 protein levels concomitant with an increase in Cyclin B (Figure 2A), a reduction of G1/G0 cells, and an increase in G2/M cells (Figure 2B,C). Together these results suggest that NRF2 propels cell cycle progression.

### 3.2. NRF2 Absence Delays G1 Exit

**Figure 3:**
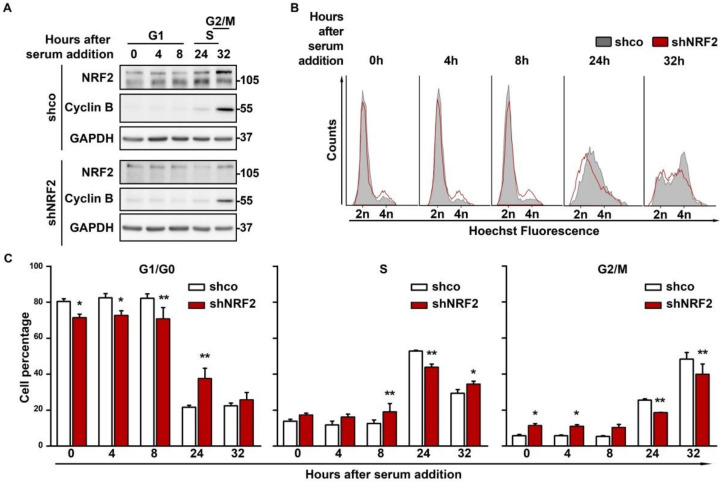
NRF2 absence reduces proliferation rate through an increase in G1 duration. U–373 MG cells were transduced with lentiviral vectors containing control (shco) or shNRF2–1 (shNRF2). Cells were then serum–deprived for 64 h for G1/G0 synchronization and then replated in serum–containing medium for cell cycle release. (**A**) Representative immunoblots of NRF2, Cyclin B, and GAPDH as a loading control; (**B**) Changes in cell cycle distribution between G1/G0, S, and G2/M phases after G1/G0 synchronization were determined by Hoechst staining (a.u., arbitrary units of fluorescence emission). A representative sample of 10,000 cells is shown for each condition; (**C**) Quantification of cells in G1/G0, S, and G2/M phases in Hoechst–stained cells. Data are presented as mean ± S.D. ** *p* ≤ 0.01; * *p* ≤ 0.05 according to a two–way ANOVA test (*n* = 3).

To determine if differences in the proliferation between shco and shNRF2 cells are due to a restriction in transit towards the S phase, we synchronized them in G1/G0 by 64 h of serum deprivation, followed by a change to serum–containing medium for cell cycle re–entry. The NRF2 knock–down was confirmed by immunoblot analysis, and Cyclin B levels were used as a control of cell cycle progression (Figure 3A). There was a slight oscillation in the NRF2 levels, with a modest increase along G1 that was maximal at the S phase, and a slight decrease at G2/M. According to the flow cytometry profiles (Figure 3B,C), a notorious difference in cell cycle progression was observed after 24 h of serum stimulation. At this time point, 60.5% of shco cells vs. only 33.2% of shNRF2 cells had exited G1. This difference was also detected at 32 h when 48.3% of shco cells vs. 39.9% of shNRF2 cells had reached the G2/M phase.

**Figure 4:**
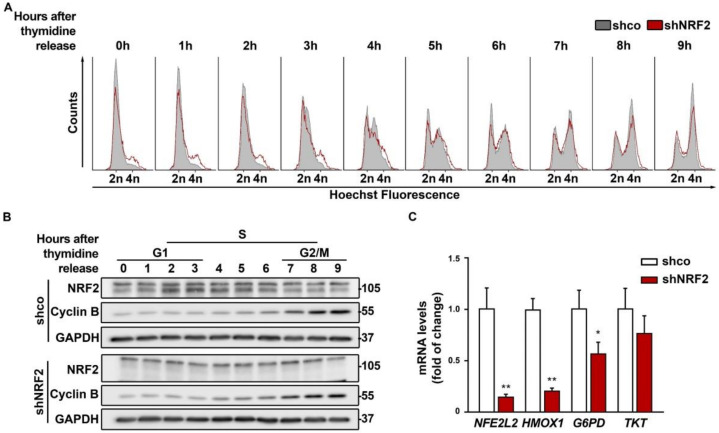
Double thymidine pulse for cell cycle synchronization indicates oscillation of NRF2 levels along the G1/S transition. U–373 MG cells were transduced with lentiviral vectors containing control (shco) or shNRF2–1 (shNRF2). Cells were synchronized before the S phase through a double thymidine pulse and then released. (**A**) Changes in cell cycle distribution between G1/G0, S, and G2/M phases after synchronization were determined by Hoechst staining (a.u., arbitrary units of fluorescence emission). A representative sample of 10,000 cells is shown for each condition; (**B**) Representative immunoblot analysis of NRF2, Cyclin B, and GAPDH as a loading control. Cell cycle phases corresponding to each time point are indicated according to the cell cycle distribution analysis of (**A**); (**C**) mRNA levels of *NFE2L2*, *HMOX1*, *G6PD*, and *TKT*, were determined by qRT–PCR and normalized by the geometric mean of *GAPDH* and *TBP*. Data are presented as mean ± S.D. ** *p* ≤ 0.01; * *p* ≤ 0.05 according to a Student’s *t*-test (*n* = 4).

In additional experiments, cells were synchronized with a double thymidine pulse and monitored for 9 h upon thymidine withdrawal (Figure 4A). Although most of the cells stayed in G0/G1 after synchronization, the shNRF2 cells again showed a modest accumulation in the G2/M phase compared to the control shco cells, which was also observed in (Figure 3B,C). Besides, there was not a clear difference in S phase entry and progression between the shco and shNRF2 cells. As shown in Figure 4B, the NRF2 protein levels increased after 2–4 h, as cells approached the G1/S transition and then slowly decreased as cells approached G2/M.

The pentose phosphate pathway (PPP) provides NADPH and nucleotide precursors, both of which are required for anabolic pathways and DNA replication of proliferating cells [2]. Considering that NRF2 is a well–known activator of genes related to the oxidative and sugar interconverting phases of the PPP, we analyzed if NRF2–depletion could affect cell cycle progression. As shown in Figure 4C, NRF2 depletion led to a significant reduction of its canonical target *HMOX1*, encoding HO–1, and to a subtle decrease in the transcript levels of glucose 6–phosphate dehydrogenase (*G6PD*) and transketolase (*TKT*) that were at the limit of statistical significance. Therefore, NRF2–depletion had a modest impact on PPP downregulation.

### 3.3. The Absence of NRF2 Delays Mitosis Withdrawal

**Figure 5:**
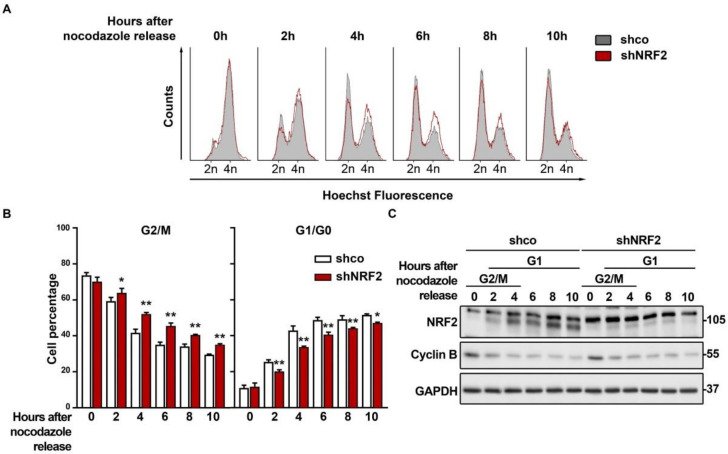
NRF2 absence delays M phase exit. U–373 MG cells were transduced with lentiviral vectors containing shcontrol (shco) or shNRF2–1 (shNRF2). Cells were then synchronized in the M phase through nocodazole treatment. Mitotic, round, partially detached cells were then harvested and replated in nocodazole–free medium for cell cycle release. (**A**) Changes in cell cycle distribution between G1/G0, S, and G2/M phases after M synchronization were determined by Hoechst staining (a.u., arbitrary units of fluorescence emission). A representative sample of 10,000 cells is shown for each condition; (**B**) Quantification of changes in G2/M and G1/G0 cell numbers. Data are presented as mean ± S.D. ** *p* ≤ 0.01; * *p* ≤ 0.05 according to a two–way ANOVA test (*n* = 3); (**C**) Representative immunoblot analysis of NRF2, Cyclin B, and GAPDH as a loading control. Cell cycle phases corresponding to each time point are shown, according to the cell cycle distribution analysis shown in (**A**).

We analyzed if, as suspected from the results obtained with the double thymidine pulse, NRF2 depletion was affecting M exit. We synchronized the shco and shNRF2 cells in the M phase with nocodazole and then released them to analyze the M–to–G1 phase transit. The G2/M cell counts indicated that the shNRF2 cells exit mitosis more slowly than the shco cells (Figure 5A,B). This observation was consistent with the reduced number of shNRF2 cells transiting throughout G1. Conversely, upon 2 h of nocodazole removal, 25.4% of shco vs. 20.1% in shNRF2 cells were in the G1 phase. This difference was maximal at 4 h (42.9% of shco cells vs. 33.7% of shNRF2 cells) and was still slightly observed by 10 h (51.6% of shco cells vs. 47.1% of shNRF2 cells). Consequently, NRF2 absence results in a delay in mitosis exit. In addition, we observed an increase in NRF2 protein levels after 2 h, concomitant with G1 entry, which reached plateau levels between 4 and 10 h (Figure 5C). These results further support the increase in NRF2 protein levels as a requisite for G1/S transition.

### 3.4. NRF2 Absence Alters Regulatory Mechanisms of G1–S, Mitosis and DNA Damage Checkpoints

**Figure 6:**
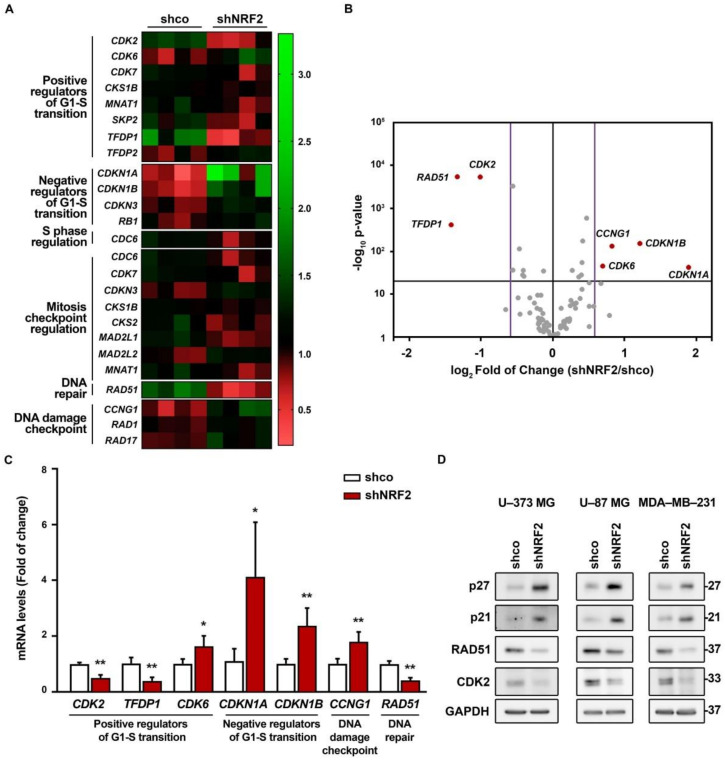
NRF2 absence alters the expression of several genes implied in cell cycle progression. U–373 MG cells were transduced with lentiviral vectors containing control (shco) or shNRF2–1 (shNRF2). (**A**) Changes in expression of 25 out of 84 different genes implied in cell cycle regulation analyzed through RT^2^ Profiler PCR Array Human Cell Cycle from Qiagen for shco and shNRF2 U–373 MG cells. The 20 genes with significant changes in their expression (*p*-value ≤ 0.05) were grouped by their biological function in a heat map, representing the individual fold of change compared to the media of all eight samples; (**B**) Volcano plot representing *p*-value (calculated through a Student’s *t*-test of shNRF2 vs. shco, *n* = 4) and fold of change for the differences in gene expression between shco and shNRF2 cells. The *p*-value threshold was established to be less than 0.05 (black horizontal line), while the fold of change threshold was established at 1.5/0.66 (purple vertical lines). Genes surpassing both thresholds are shown in red with their respective names, while the rest of the genes are represented in grey; (**C**) Changes in gene expression in genes with a *p*-value lower than 0.05 and a fold of change higher than 1.5 or lower than 0.66. Data are presented as mean ± S.D. ** *p* ≤ 0.01; * *p* ≤ 0.05 vs. shco according to a Student’s *t*-test (*n* = 4); (**D**) Representative immunoblot analysis of p21, p27, RAD51, CDK2, and GAPDH as a loading control in lysates from U–373 MG, U–87 MG, and MDA–MB–231 cells transduced with lentiviral vectors containing control (shco) or shNRF2–1 (shNRF2).

We analyzed the expression of a battery of genes involved in cell cycle regulation using a NRF2–knockdown approach and the RT^2^ Profiler PCR Array Human Cell Cycle from Qiagen (see Materials and Methods). We grouped all genes with significant expression changes (*p*-value ≤ 0.05) by their biological function in a heat map (Figure 6A). We detected a reduced expression of several G1/S progression genes (*CDK2*, *TFDP1*, *CDK7*, *CKS1B*, *MNAT1*, and *SKP2*), and upregulation of *TFDP2* and *CDK6*. There was also an upregulation of *CDKN3*, *CDKN1A*, *CDKN1B*, and *RB1*, the negative regulators of G1 exit, and downregulation of CDC6, implied in S phase progression. In addition, there were also changes in the expression of mitosis checkpoint genes (*CDC6*, *CDK7*, *CDKN3*, *CKS1B*, *CKS2*, *MAD2L1*, *MAD2L2*, and *MNAT1*), and an upregulation of DNA damage checkpoint–related genes *RAD1*, *RAD17*, and *CCNG1*, and DNA repair *RAD51*. Then, we further analyzed the gene expression changes with a threshold *p*-value of 0.05 but with a restrictive fold of change of 1.5/0.66 (Figure 6B). We still detected seven different genes with altered expression (Figure 6B,C). This restrictive analysis suggested a need for NRF2 in the expression of positive regulators of G1–S transition (*CDK2*, *TFDP1*) and repression of negative regulators of G1–S transition (*CDKN1A* and *CDKN1B*). Importantly, NRF2 appears to be needed for optimal expression of DNA damage detection (*CCNG1*) and repair (*RAD51*). Some of these genes were further analyzed by immunoblot in U–373 MG, U–87 MG, and MDA–MB–231 cells (Figure 6D), further confirming the upregulation of p27 (*CDKN1B*), p21 (*CDKN1A*), and the downregulation of CDK2 and RAD51 in the three cell lines. Altogether, these results provide additional information about the participation of NRF2 in controlling the regulation of G1/S and mitotic checkpoints.

**Figure 7:**
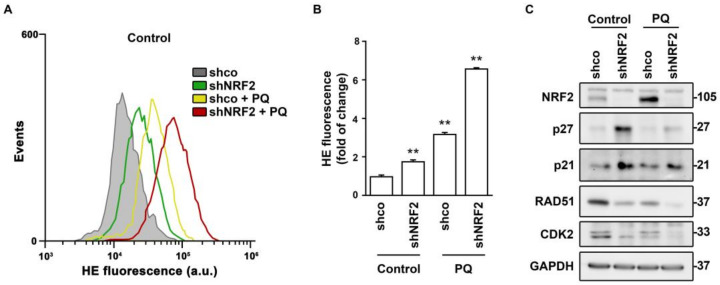
Regulation of cell cycle effectors by NRF2–deficiency and paraquat–induced redox dysregulation. U–373 MG cells were transduced with lentiviral vectors containing control (shco) or shNRF2–1 (shNRF2) and treated with vehicle or 300 µM PQ for 48 h. (**A**,**B**) Flow cytometry analysis of intracellular ROS production in hydroethidine (HE) stained cells. A representative sample of 10,000 cells is shown for each condition. Data are presented as mean ± S.D. ** *p* ≤ 0.01 according to a one–way ANOVA test (*n* = 3); (**C**) Representative immunoblot analysis of NRF2, p27, p21, RAD51, CDK2, and GAPDH as a loading control.

Considering that NRF2 is a master regulator of redox homeostasis, the changes in these cell cycle regulators might be redox–dependent. This hypothesis was tested in the presence of the pro–oxidant paraquat (PQ) (Figure 7). Both NRF2 absence and PQ treatment induced ROS accumulation (Figure 7A,B). NRF2 depletion led to the expected increase in p21 or p27 levels, but the enhanced ROS accumulation induced by PQ did not further increase, or even decreased, p21 or p27 levels (Figure 7C). By contrast, the RAD51 and CDK2 levels were reduced in shNRF2 cells and exhibited a further reduction in the presence of PQ. Consequently, changes in these cell cycle effectors in the absence of NRF2 seem to respond to different mechanisms, with RAD51 and CDK2 showing the best correlation with redox alterations.

## 4. Discussion

While most of the effects of NRF2 on proliferation have been analyzed in the presence of NRF2 activators and aimed at elucidating its participation in tumorigenesis, its participation in cell cycle progression was little explored. To address this issue, first, we analyzed the effect of NRF2 on cell cycle phase distribution and proliferation rate. Three different cell lines, U–373 MG, U–87 MG, and MDA–MB–231 yielded essentially similar responses to NRF2 depletion, increasing G1/G0 and decreasing G2/M cell counts, and slowing down proliferation. In other words, NRF2 depletion tends to stop cell cycle progression. These results are consistent with reports showing that the silencing of the NRF2 coding gene is associated with the induction of premature senescence [22].

NRF2 depletion led to an accumulation of G1 cells indicating that they cannot efficiently pass the G1/S restriction point. Besides, NRF2 levels seem to be maximal at S phase entry, suggesting that NRF2 deficiency prevents the proper preparation of cells to enter the S phase. This hypothesis is consistent with the observation that NRF2–deficiency led to a reduced expression of the positive cell cycle regulators *CDK2* and *TFDP1* and increased expression of the cell cycle inhibitors, *CDKN1A* and *CDKN1B*, encoding p21^waf^ and p27^kip1^, respectively.

The cell cycle kinase inhibitor p21 participates in cell cycle regulation, DNA repair, and oxidative stress response [23]. It has been reported that NRF2 transcriptionally activates p21 and promotes cell defenses against oxidative stress induced by hydrogen peroxide [24]. However, p21 also participates in the onset of cellular senescence, suggesting the need for a tight control regulation of its function [25]. p21 is an effector of the tumor suppressor protein p53, which is involved in the regulation of the cell cycle and DNA repair and is activated by oxidative stress [26,27]. P53 activation leads to transient expression of p21, triggering transient G1 cell cycle arrest [28]. Nevertheless, among the cell lines studied, U–373 MG cell line is known to have a mutated p53, suggesting that under our experimental conditions, p21 upregulation is p53 independent. Further work is required to determine the crosstalk among NRF2, p53, and p21 in the regulation of G1/S transition, but our results are consistent with a role of NRF2 in propelling cells through this checkpoint.

NRF2–knockdown also led to the upregulation of the CDK inhibitor p27 (*CDKN1B*). NRF2 deficiency has been reported to decrease the p27 levels in response to Angiotensin II in cardiomyocytes [29]. This effect was not due to the direct inhibition of *CDKN1B* and therefore it might represent a post–translational effect of Angiotensin II activity. In fact, our results are in line with [16], in which depletion of *PERK1*, a kinase that activates NRF2 under stress conditions, led to the downregulation of p27 and p21. It is most likely that the difference among these studies is related to the dual role of CDK inhibitors, stopping the cell cycle until cells are ready for S phase entry or leading to senescence if there is a persistent impediment [30,31].

NRF2–depletion also led to a reduced transit throughout G2/M. Our results are consistent with a previous study which reported that Nrf2–deficient primary epithelial cells exhibit redox alterations, and G2/M–phase arrest that could be partially reversed by treatment with N–acetylcysteine and glutathione [32]. We found a role of NRF2 in the control of DNA integrity. *RAD51* participates in the homologous recombination and DNA repair, while *CCNG1* is associated with G2/M phase arrest in response to DNA damage. Therefore, our findings are consistent with a deficiency in the surveillance of DNA integrity and timely repair, leading to the extension of G2/M duration. In agreement with our results, it has been previously reported that NRF2 facilitates the repair of radiation–induced DNA damage through the induction of *RAD51* [8,33]. The authors identified putative NRF2–responsive enhancers in *RAD51* and other repair genes that are involved in the homologous recombination repair pathway. However, our analysis of the Encyclopedia of DNA Elements (ENCODE) of the human genome failed to retrieve a significant site for NRF2 binding, or for its partner MAFK, to the promoter of *RAD51* (data not shown).

Considering the need for NADPH in cycling cells for anabolic reactions and redox control, we explored the effect of NRF2–deficiency in the expression of two limiting genes of the oxidative and sugar converting phases of the pentose phosphate pathway (PPP), glucose 6 phosphate dehydrogenase (*G6PD*) [34], and transketolase (*TKT*) [35], which are regulated by NRF2 [2]. The transcript levels of *G6PD* and *TKT* were very little changed after NRF2 depletion, consistent with our previous results that the ROS levels of this cell line are low and insensitive to NRF2 modulation [13].

Nocodazole inhibits microtubule polymerization and therefore arrests cells at mitosis. Upon nocodazole withdrawal, the NRF2–depleted cells exhibited a delay in mitosis exit, suggesting that, even though NRF2 levels are low at mitosis, it exerts still unexplored effects on the transition through this cycle phase. However, we also detected reduced NRF2 levels in G2/M in cells synchronized with the double thymidine pulse. Then, considering the reduced NRF2 levels and chromatin condensation that would further limit its transcriptional activity, the role of NRF2 in this cell cycle phase may be transcription–independent. The transcription–independent effects of NRF2 have already been postulated in the widely used Nrf2–knockout mice, which expresses a fusion protein of N–terminal NRF2 bound to LacZ [36]. In these mice, NRF2 still serves as a protein tethering KEAP1, and, consequently, a reduction of NRF2 levels would lead to KEAP1 release for the degradation of other proteins [37]. The role of KEAP1 in the cell cycle has been reported for p21 in the G1/S transition [38,39] but its role in mitosis has not been reported yet. Considering that NRF2 depletion, and the consequent increased KEAP1 activity, leads to delayed mitosis, it is interesting to speculate that KEAP1 could somehow antagonize the proteolytic effect of Anaphase–Promoting Complex/Cyclosome (APC/C), which participates in the destruction of cycle regulators, including securing B type cyclins, to propel metaphase to anaphase transition [40].

NRF2 protein levels oscillate during cell cycle progression, being maximal at the G1/S, and minimal at the G2/M restriction points. NRF2 levels are regulated through a combination of modifications in its gene expression by specific transcription factors and epigenetics, as well as by modification of protein stability by the ubiquitin–proteasome system. Further work is required to identify the mechanisms that govern fluctuations in NRF2 levels and activity along the cell cycle.

## Figures and Tables

**Table 1:** Oligonucleotides used for qRT–PCR.

Gene	Forward Sequence 5′–3′	Melting Temp. (°C)	Reverse Sequence 5′–3′	Melting Temp. (°C)
*GAPDH*	CTCTCTGCTCCTCCTGTTCGAC	66.7	TGAGCGATGTGGCTCGGCT	71.9
*TBP*	TGCACAGGAGCCAAGAGTGAA	68.3	CACATCACAGCTCCCCACCA	69.8
*NFE2L2*	AAACCAGTGGATCTGCCAAC	63.9	GTGACTGAAACGTAGCCGAAGA	65.4
*HMOX1*	TGCTCAACATCCAGCTCTTTGA	67.1	GCAGAATCTTGCACTTTGTTGC	66.5
*G6PD*	TGACCTGGCCAAGAAGAAC	64.9	CAAAGAAGTCCTCCAGCTTG	61.6
*TKT*	ACATCTACCAGAAGCGGTGC	64.2	TTCTACCCCCGTGATCCCTC	67.2

## Data Availability

Data are available upon request to the corresponding author.

## Supplementary Materials

The following supporting information can be downloaded at: https://www.mdpi.com/article/10.3390/antiox11050946/s1. Figure S1: NRF2 increases G1/G0 cell fraction and reduces cell proliferation rate; Figure S2: NRF2 downregulation increases G1 cell numbers.
