# Supplementary material for: Transcription Factor NRF2 Participates in Cell Cycle Progression at the Level of G1/S and Mitotic Checkpoints
Source: Antioxidants (Basel). 2022 May 11;11(5):946. doi: 10.3390/antiox11050946 (PMC9137878; doi:10.3390/antiox11050946)
Supplement: Supplementary file 1 [file antioxidants-11-00946-s001.zip › antioxidants-1670614-supplementary.pdf]

antioxidants

MDPI

Supplementary Materials

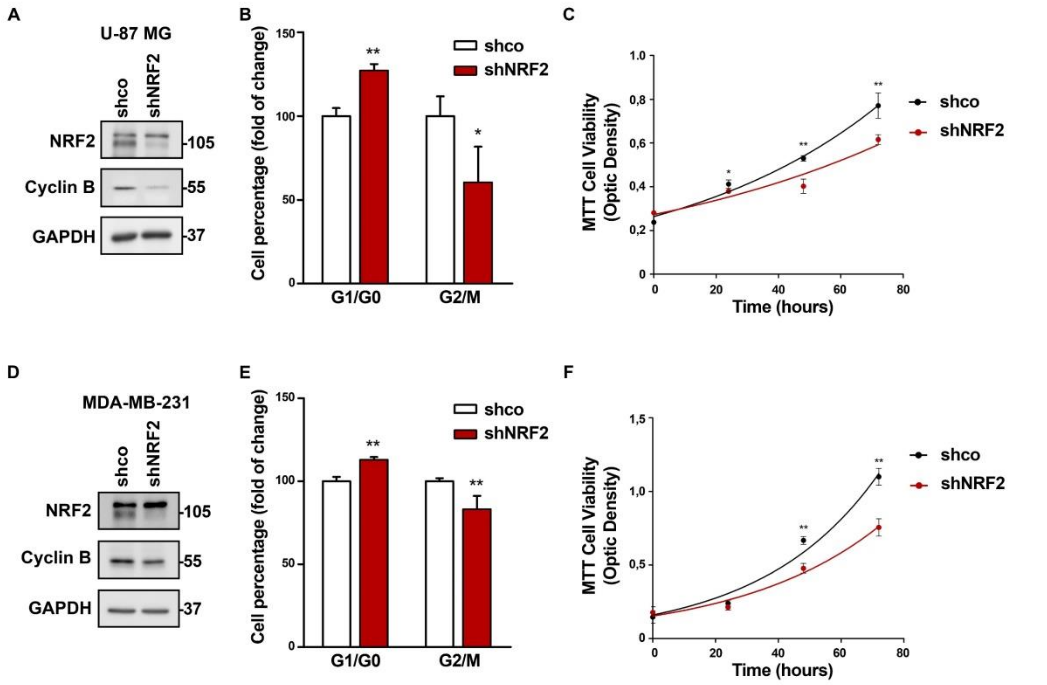

Figure S1. NRF2 increases G1/G0 cell fraction and reduces cell proliferation rate. **(A–C)** U-87 MG glioblastomas cells. **(D–F)** MDA-MB-231 breast adenocarcinoma cells. Both cell lines were transduced with lentiviral vectors containing control (shco) or shNRF2-1 (shNRF2). **(A,D)**, Representative immunoblots of NRF2, Cyclin B, and GAPDH as a loading control. **(B,E)** Flow cytometry analysis of changes in cell cycle distribution in Hoechst-stained cells. Data are presented as mean ± S.D. ** $p \leq 0.01$; * $p \leq 0.05$ according to a Student's t-test (n = 3). **(C,F)** Proliferation rate in shNRF2 cells based on MTT viability assay. The curve was fitted according to an exponential growth model with the least square fit. Data are presented as mean ± S.D. ** $p \leq 0.01$; * $p \leq 0.05$ vs. shco according to a two-way ANOVA test (n = 4).

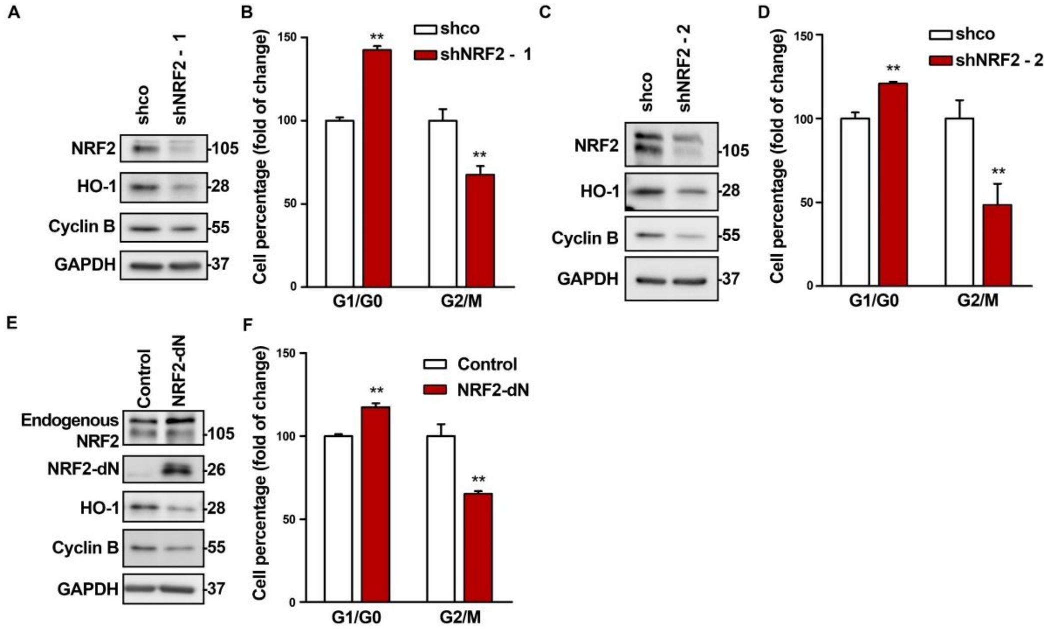

Figure S2. NRF2 downregulation increases G1 cell numbers. U-373 MG cells were transduced with different lentiviral vectors **(A,B)** Knockdown of NRF2 with lentiviral vectors containing control (shco) or shNRF2-1. **(C,D)** Knockdown of NRF2 with lentiviral vector containing control (shco) or

shNRF2-2. For A and C, representative immunoblots of NRF2, HO-1, Cyclin B, and GAPDH as a loading control. For B and D, flow cytometry analysis of G1 and G2/M phases in Hoechst-stained cells. Data are presented as mean ± S.D. ** $p \leq 0.01$ according to a Student's t-test ($n = 3$). **(E,F)** U-373 MG glioblastomas cells were transduced with lentiviral vectors containing NRF2 dominant-negative mutant (NRF2-dN) or an empty vector (Control). **(E)** Representative immunoblots of NRF2, HO-1, Cyclin B, and GAPDH as a loading control. **(F)** Flow cytometry analysis of G1 and G2/M phases in Hoechst-stained cells. Data are presented as mean ± S.D. ** $p \leq 0.01$ according to a Student's t-test ($n = 3$).
